# Optical Limiting in a Novel Photonic Material—DNA Biopolymer Functionalized with the Spirulina Natural Dye

**DOI:** 10.3390/molecules30234577

**Published:** 2025-11-28

**Authors:** Petronela Gheorghe, Adrian Petris

**Affiliations:** National Institute for Laser, Plasma and Radiation Physics, 409 Atomistilor Street, 077125 Magurele, Romania

**Keywords:** DNA biopolymer, Spirulina, I-scan, optical nonlinearities, optical limiting

## Abstract

The results of an experimental comparative study on absorptive nonlinear optical properties of deoxyribonucleic acid (DNA)–cetyltrimethylammonium chloride (CTMA) biopolymer functionalized with spirulina natural dye, as solutions in butanol, and on the same nonlinear optical properties of similar solutions with spirulina only, are presented. The spectroscopic characterisation of the investigated complexes is performed by Ultraviolet–Visible-Near-Infrared (UV-VIS-NIR) spectroscopy and Attenuated Total Reflection Fourier-transform Infrared (ATR-FTIR) spectroscopy. Their optical limiting functionality is experimentally demonstrated at the wavelength of 1550 nm (an important telecommunication wavelength) using ultrashort laser pulses (~120 fs). Important parameters that characterise the optical limiting (nonlinear absorption coefficient *β*, and saturation intensity, *I*_sat_) are determined by the Intensity-scan (I-scan) method in the investigated materials. The results of our experimental investigation reveal, for the first time to the best of our knowledge, a significant absorptive nonlinear optical response of spirulina natural dye and its potential for optical limiting. The favourable effect of the DNA biopolymer on the nonlinear optical response of the investigated solutions, resulting in the enhancement of their nonlinear optical properties, is demonstrated. Thus, the investigated DNA–CTMA–spirulina liquid compound is a promising novel “green” material for passive optical limiting devices to protect sensitive optical and optoelectronic devices from high-intensity near-infrared laser beams. Also, from dye-doped DNA compounds as solutions it is possible to obtain, by different methods (e.g., spin-coating, drop casting), thin films as the base of all-optical solid-state limiting devices.

## 1. Introduction

Strongly stimulated by the development of high-power lasers, nonlinear optics is an important and rapidly evolving field of modern optics with numerous applications based on the nonlinear optical response of materials to laser light excitation [1].

Passive optical limiting functionality, based exclusively on the nonlinear absorption process, is currently being intensively studied [2]. The main application of optical limiting functionality is to protect the human eye, light sensors, cameras, and other sensitive optical and optoelectronic devices against intense sources of laser radiation, which can irreversibly damage them when a safety damage threshold is exceeded [3,4,5,6,7,8,9]. The evolution of this topic is a direct consequence of the rapid development of laser technology, particularly of the pulsed lasers with ultrashort and ultra-intense light pulses.

**Figure 1:**
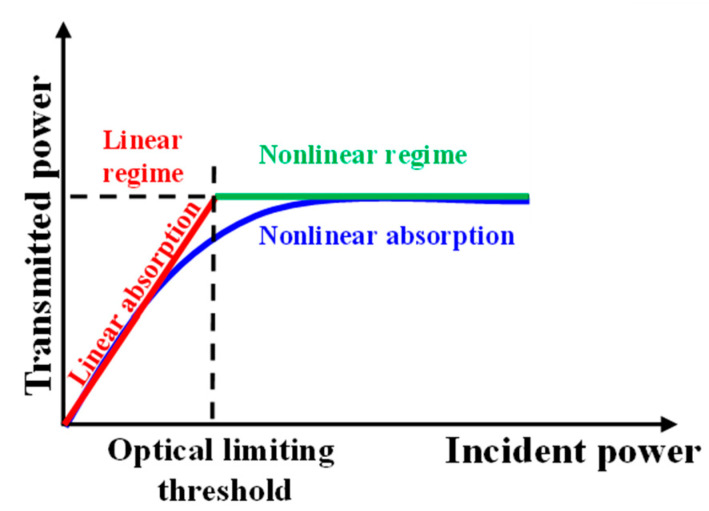
Optical response of an optical limiter.

Passive optical limiting is a nonlinear optical phenomenon that occurs in materials that exhibit large absorptive optical nonlinearities, as graphene-based materials [10,11,12], fullerenes [13], carbon nanotubes [14], synthetic and natural dyes and compounds containing them [15,16,17,18,19]. The transmittance of an optical limiting material remains constant for incident intensities lower than a certain threshold, and it decreases when the incident light intensity is above this threshold, maintaining a constant value of the transmitted light intensity. A schematic of the optical response of an optical limiter is shown in Figure 1.

In a real optical limiter (Figure 1, blue line), there is a continuous transition from the linear absorption regime (constant transmittance) to the nonlinear absorption regime (decreasing transmittance), whereas in an ideal one, there is a clearly defined threshold between the linear and the nonlinear regime of transmittance, as is shown in Figure 1.

Recently, a new class of optical materials based on the DNA biopolymer [20,21,22] attracted a lot of interest in organic photonics (ultrafast nonlinearities [23], laser-induced dynamic gratings [24,25], all-optical spatial phase modulation [26,27], lasing [28,29]) and electronics [30,31,32]. DNA is an eco-friendly material, which is usually extracted from the waste of the food processing industry, a renewable resource. Unlike synthetic polymers, the DNA biopolymer is biodegradable, which is an advantage in reducing environmental pollution and sustainable development [31]. Moreover, when this biopolymer is functionalized with natural dyes (e.g., spirulina), it becomes even more attractive for applications in photonics [8,15,33]. Therefore, it is worth exploring the absorptive optical nonlinearities of both spirulina natural dye alone and of the DNA–CTMA–spirulina compound to assess their optical limiting performance. Pure DNA is water-soluble only, but this property negatively affects its use in photonics. With certain surfactants such as CTMA, DNA forms water-insoluble complexes (DNA–CTMA) with a much higher stability. On the other hand, DNA–CTMA is soluble in many organic solvents (e.g., different alcohols such as ethanol, butanol, isopropanol), facilitating its functionalization with different dyes [20,21,33,34,35]. From dye-doped DNA solutions, it is possible to obtain, by various methods (e.g., spin-coating, drop casting), thin films [24,26,36,37] as the base of all-optical solid-state photonic devices. Some important properties of DNA that make it suitable as an optical material are briefly discussed below.

DNA is highly transparent in visible (VIS) and near-infrared (NIR) spectral domains, a property which is not altered by the CTMA surfactant. The specific double-stranded structure of the DNA molecule can ensure a larger free volume for doping photosensitive molecules than synthetic polymers and a protective effect against photo-degradation, also [23,27,28,31,36,38]. Thus, a faster and enhanced nonlinear optical response to light excitation can be obtained in dye–DNA compounds compared to that in dye–synthetic polymer compounds [21,23,37,39]. A laser beam that interacts with a material inherently heats it, more or less, depending on the laser beam characteristics and on the absorption and thermal properties of the material. This heating usually has a detrimental effect on many photonic applications. Since the local laser-induced increase in the temperature of an optical material is inversely proportional to its thermal conductivity, the optical materials with high values of this parameter are preferable. When comparing the value of the thermal conductivity of the synthetic polymers as, e.g., polymethylmethacrylate (PMMA) (0.12 W/(m·K)), polyethylene glycol (PEG) (0.19–0.32 W/(m·K)) with the values of this parameter for DNA (0.82 W/(m·K)) and of DNA–CTMA (0.6 W/(m·K)), which are several times higher, a clear advantage of using optical materials based on the DNA biopolymer results [22,39,40,41]. The threshold for laser-induced damage is higher in DNA and DNA–CTMA than that in some synthetic polymers, such as PC or PEG [15,31,42].

By functionalizing the DNA with different dyes, DNA-based compounds with optical properties suitable for various photonic applications can be made.

Spirulina natural dye can be easily obtained at low cost from its commercially available powdered form. Being biodegradable and environmentally safe, it represents an attractive choice for making fully biodegradable compounds with the DNA biopolymer. Both spirulina and DNA are highly transparent in visible and near-infrared spectral domains [43,44], an important property for their use in photonics as “green” optical materials. From compound solutions of DNA–CTMA–spirulina in different alcohols, it is possible to obtain films which are attractive for all-optical solid-state photonic devices. The investigation of the nonlinear optical response in NIR, at 1550nm, of DNA–CTMA–spirulina is important for the assessment of the possibility of using this compound for photonic applications.

For the experimental investigation of the nonlinear response of nonlinear optical materials, several methods, such as Z-scan [45,46,47,48,49], wave-mixing, pump-probe interferometry [26,27,50], and third harmonic generation [37], have been developed.

Z-scan and the method derived from it, I-scan, are simple single-beam sensitive optical methods for measuring the magnitude and the sign of the nonlinear refractive index and of the nonlinear absorption coefficient of transparent materials. The Z-scan method was introduced by M. Sheik-Bahae, A. A. Said, and E. W. Van Stryland [47] in 1990. In this method, the sample is moved along the propagation direction of the laser beam (*z*-axis), passing through the focal plane of a focusing lens. This action leads to a change in the intensity of excitation with the distance from the focus during movement. The changes in the optical phase/transmittance are monitored in the far field with a detector in front of which a small aperture is placed (closed-aperture Z-scan, refractive nonlinearities), or by collecting the entire transmitted beam (open-aperture, absorptive nonlinearities) [47].

I-scan is an alternative method [48], derived from the Z-scan method, and has several advantages over it in some experiments. In Z-scan, during the movement of the sample along the direction of light propagation (*z*-axis), the size of the illuminated area of the sample depends on its position relative to the focal plane of the focusing lens, and the collected signal is averaged over areas of different sizes. This could be a problem in the case of inhomogeneous samples, or for small-sized samples, for which the laser beam spot is smaller than the sample, only around the focal plane of the focusing lens. In I-scan, the sample position is fixed relative to that of the focusing lens. The size of the illuminated spot on the sample is constant, and the intensity of the beam incident on the sample is changed by using optical attenuators. The lack of moving elements in the experimental setup leads to a considerable minimisation of the misalignment errors introduced by optical components. When investigating sensitive samples with an unknown threshold for laser-induced damage, placing the samples in the focal plane of the focusing lens should be avoided. This is another advantage of this method, because the samples no longer pass through the focal point of the focusing lens, as in the Z-scan method, thus reducing the risk of their damage. Moreover, the total exposure time of the sample is reduced, so other changes in the sample, such as thermal effects, are smaller.

In this work, we investigated for the first time, to the best of our knowledge, the passive optical limiting in spirulina natural dye and in the DNA biopolymer functionalized with spirulina dye of different concentrations. The nonlinear absorption was experimentally investigated with near-infrared ultrashort laser pulses (~120 fs) at the important telecommunication wavelength of 1550 nm. The nonlinear absorption coefficient, *β*, and the saturation intensity, *I*_sat_, have been determined in the investigated materials to assess the potential of spirulina dye only and of DNA–CTMA-spirulina compound for optical limiting and the effect of DNA on the nonlinear optical response of the investigated solutions.

The two components, spirulina natural dye and DNA–CTMA bio-polymer, have a synergic effect in the compound investigated in this paper, based on the absorptive nonlinear response of spirulina and on the protective environment ensured by DNA–CTMA [15]. Thus, the optical limiting functionality of the novel DNA–CTMA–spirulina nonlinear compound is improved compared to that of spirulina alone.

## 2. Materials and Methods

### 2.1. Preparation of the Investigated Samples

The investigated samples are DNA–CTMA–spirulina solutions of different concentrations in butanol, and spirulina solutions in butanol (Merck KGaA, Darmstadt, Germany) with the same concentrations. The DNA used in our samples was purchased from Ogata Research Laboratory, Ltd., Chitose, Japan, and used as it was purchased. It was extracted from salmon waste produced by the food processing industry.

The DNA was functionalized with the CTMA surfactant (Merck KGaA, Darmstadt, Germany) to obtain a water-insoluble complex. The procedure followed for this is the one described by Grote et al. [51]. This method consists of the dropwise addition of the DNA solution to the CTMA solution. The obtained DNA–CTMA complex is filtered, dried in an oven at 40 °C for 24 h and ground to facilitate its dissolution in butanol. The preparation of the DNA–CTMA complex involves an ion-exchange reaction, where purified DNA in aqueous solution is precipitated using the cationic surfactant CTMA. During this process, positively charged CTMA reacts with negatively charged DNA through an ionic interaction. The resulting complex is water-insoluble, with an enhanced chemical and mechanical stability due to the long hydrophobic alkyl chain of the surfactant. Once formed, the DNA–CTMA complex becomes soluble in a range of organic solvents such as ethanol, methanol, and butanol. This solvent compatibility allows the incorporation of various synthetic and natural dyes into the DNA–CTMA matrix, leading to the desired spectral sensitivity of the compounds. Furthermore, the double-helical structure of DNA–CTMA offers a larger free volume for guest molecules in comparison with synthetic polymers, making it a suitable host for photoactive molecules and facilitating fast photo-induced effects in dye-doped DNA-based materials [23,26].

The raw spirulina used in our experiments was acquired as 100% pure powder of ecological origin from a health food store. The natural extract of spirulina was obtained by maceration, a simple method for obtaining the natural extract. In this powder form, the contact surface of the raw material with the solvent (butanol) is as large as possible. The solvent was added over the powder and left in contact for 72 h. During this period, the spirulina–butanol mixture was placed on a magnetic plate (400 rpm) for stirring to obtain a homogeneous complex. After this time, it was filtered and kept in a refrigerator in dark glass containers to avoid degradation of the active molecules and to prevent the development of micro-organisms. For the functionalization of the DNA–CTMA biopolymer with the spirulina natural dye, we used butanol as an organic solvent, which has several advantages over other solvents. It has a lower vapour pressure than, e.g., ethanol [52] and a moderate viscosity, resulting in slower evaporation [53].

In all prepared solutions of DNA–CTMA–spirulina, the concentration of DNA–CTMA in butanol was 30 g/L. Five solutions of DNA-CTMA-spirulina in butanol with a concentration of spirulina dye ranging from 3 g/L to 15 g/L were investigated. Also, for comparison, five solutions of spirulina only in butanol, with the same concentrations, were investigated to assess how the DNA–CTMA affects the absorptive properties of spirulina dye, and, consequently, its optical limiting functionality. Table 1 summarises all investigated samples. The denominations of the investigated samples (S3 to S15 and S3-DNA to S15-DNA) are shown in the first column, and the spirulina concentrations (g/L) are shown in the second column of the table.

**Figure 2:**
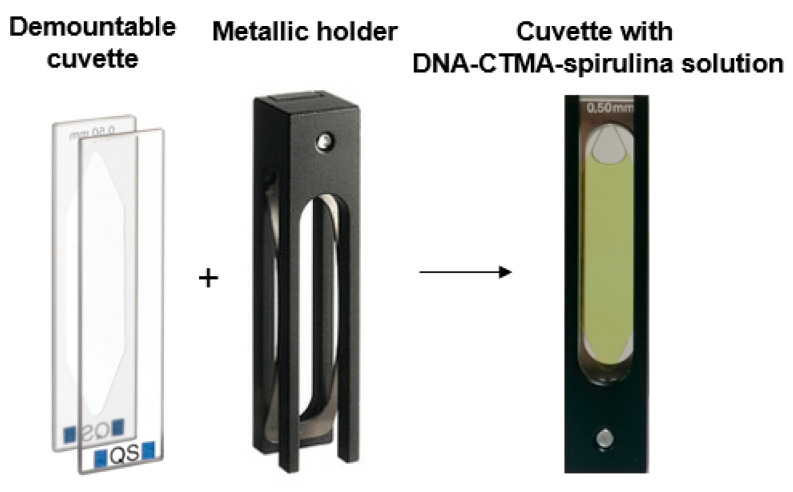
The demountable cuvette used in our experiments.

The investigated solutions were placed in special demountable cuvettes (Hellma, Müllheim, Germany) of internal thickness *L* = 0.5 mm and 130 μL internal volume, with optical quality windows made from quartz. Their spectral domain of transparency was 200 nm–2500 nm. The cuvettes were composed of two detachable parts, which were fixed in an elastic metallic mount for sealing (Figure 2). This special construction of the cuvettes, which allowed them to be disassembled by separating their two parts, ensured a facile filling and cleaning of their internal faces.

### 2.2. Linear Optical Properties

#### 2.2.1. Refractometry and UV–VIS–NIR Spectroscopy

The linear refractive index, *n*_0_, and the absorbance spectra of the prepared solutions have been measured to evaluate the linear optical properties of the investigated samples.

For the measurement of the linear refractive index, *n*_0_, a Carl Zeiss ABBE refractometer, Type-G model (Jena, Germany) was used. The values of the refractive index are slightly dependent on spirulina concentration. Thus, the solution with spirulina only of the lowest concentration had a refractive index of *n*_0_ = 1.3895, while the refractive index of the samples with DNA–CTMA–spirulina and the same concentration of spirulina was *n*_0_ = 1.4009. The refractive index of the solution with the highest concentration of spirulina was *n*_0_ = 1.3899 for samples with spirulina only, and *n*_0_ = 1.4011 for the samples DNA–CTMA–spirulina. The refractive indices of solutions with intermediate concentrations were between the values corresponding to solutions with the lowest and highest spirulina concentrations, respectively.

**Figure 3:**
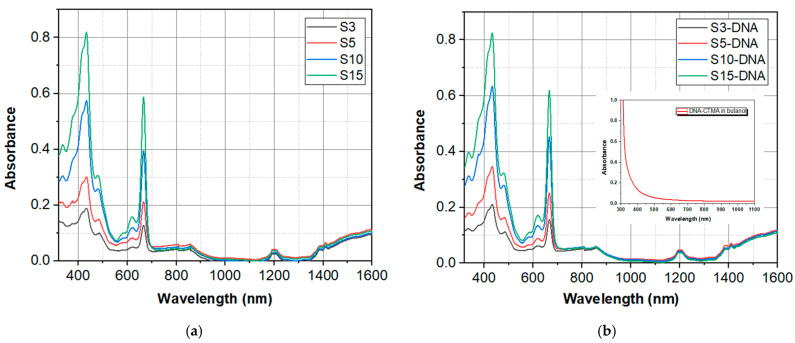
The UV-VIS-NIR absorption spectra of (**a**) spirulina alone solutions in butanol, at different concentrations and (**b**) DNA–CTMA–spirulina solutions in butanol at the same spirulina concentrations (inset—the DNA–CTMA solution in butanol).

The UV-VIS-NIR absorption spectra of the investigated samples were obtained using a Perkin Elmer spectrophotometer, Lambda 950 model (Waltham, MA, USA), at a resolution of 1 nm. The spectra were recorded for a wide range of wavelengths, between 300 nm and 1600 nm, and collected with the air as reference. The UV-VIS-NIR absorption spectra of spirulina alone solutions in butanol, at different concentrations, and of DNA-CTMA-spirulina solutions in butanol, at the same spirulina concentrations, are shown in Figure 3a and Figure 3b, respectively. In the inset in Figure 3b, the absorption spectrum of the DNA–CTMA solution in butanol is shown.

Two pronounced absorption peaks of Spirulina solutions in butanol and DNA–CTMA–spirulina solutions in the same solvent, shown in Figure 3a,b, are present in the range of visible light (400 nm to 700 nm). Their magnitude increases with the spirulina concentration. These are followed by two other smaller absorption peaks in the NIR range, at 1200 nm and 1400 nm. The small sharp decrease in absorbance immediately after 800 nm wavelength is due to the change in the light source in the spectrophotometer and is therefore not a real one.

For wavelengths longer than ~1350 nm, there is an increasing trend of absorption until 1600 nm, but the absorption in this region, which includes the 1550 nm wavelength at which the optical limiting is investigated, remains much lower than the absorption peaks from the VIS range.

As the absorption in DNA–CTMA in butanol is at wavelengths from the UV spectral range (λ < 400 nm), as can be seen from the inset in Figure 3b, the absorption in spirulina dye [51] is responsible for the features of the spectra from Figure 3a,b.

Another important optical parameter, the linear absorption coefficient, *α*_0_, of the prepared solutions, has been derived as a result of the investigation of the nonlinear optical properties by the I-scan method, which is described in Section 2.3.

#### 2.2.2. FTIR Spectroscopy

**Figure 4:**
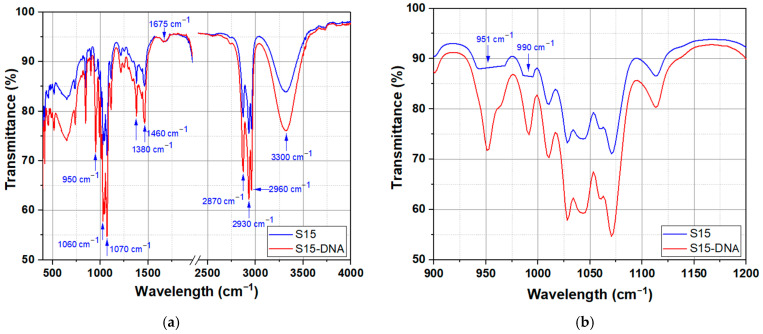
(**a**) The ATR–FTIR spectra of the spirulina solution in butanol (sample S15) and DNA–CTMA–spirulina solution in butanol (sample S15-DNA) in the wavenumber range 400–4000 cm^−1^; (**b**) The ATR–FTIR spectra of the spirulina solution in butanol (S15) and DNA–CTMA–spirulina solution in butanol (S15-DNA) at a widened horizontal scale (900–1200 cm^−1^).

FTIR spectroscopy enables the analysis of the chemical structure of the solution in butanol of spirulina and DNA–CTMA–spirulina, respectively, by identifying functional vibration groups based on their absorption of IR light. The chemical composition and molecular structure of the prepared samples were investigated by ATR-FTIR spectroscopy. The spectra of the samples, recorded using a Shimadzu IR Tracer-100 spectrophotometer (400–4000 cm^−1^ wavenumber range, 4 cm^−1^ resolution, 200 scans per spectrum), are shown in Figure 4a,b.

The broad band identified in both samples in the 3200–3500 cm^−1^ region corresponds to O–H symmetric and asymmetric stretching vibrations mainly associated with butanol and residual water [54,55], while the two strong bands observed at ~2960–2870 cm^−1^ are assigned to symmetric and asymmetric vibrations of the methylene (CH_2_) and methyl (CH_3_) groups from both butanol and CTMA surfactant [55,56]. The more pronounced peaks for the DNA–CTMA–spirulina sample in butanol are due to the contribution of these groups from both the butanol and the CTMA surfactant. The interval marked in Figure 4a (1966–2400 cm^−1^) contains the diamond lines coming from the crystal of the ATR module [57,58] and has been removed from the graph. The peak at ~1675 cm^−1^ is attributed to the C=O stretch of amides, characteristic of amide bands in proteins, which clearly confirms the presence of spirulina in the investigated samples [59,60,61]. The presence of long alkyl chains in the CTMA surfactant is further evidenced by the CH_2_/CH_3_ bending vibration bands between ~1465–1380 cm^−1^, which are more pronounced in samples containing butanol and CTMA, indicating ordered cetyl groups. These vibrations overlap with the CH_2_/CH_3_ vibrations of butanol. This is the reason why a weaker peak is also observed in the spirulina sample without DNA–CTMA.

The intense C–O stretch vibration of butanol (1060–1070 cm^−1^) overlaps with the symmetric PO_2_ stretch vibration of DNA. In the region of 951–990 cm^−1^, the presence of phosphate groups (O–P–O) assigned to the DNA backbone is evident only in the DNA–CTMA–spirulina samples (Figure 4b), which confirms the presence of DNA [55].

The presence of several diagnostic features in the FTIR data, namely the phosphate stretching modes of DNA, the amide band of spirulina proteins, and the CH_2_/CH_3_ vibrations of CTMA, confirms the presence of the DNA–CTMA–spirulina complex in butanol and of spirulina in butanol, respectively.

### 2.3. Nonlinear Optical Properties Investigated by the I-Scan Method

The passive optical limiting in a nonlinear optical material is a consequence of its absorptive nonlinearity. The optical limiting potential of the novel DNA biopolymer functionalized with spirulina natural dye was investigated by us using the I-scan method, in the NIR spectral range, at 1550 nm wavelength, with ultrashort laser pulses.

**Figure 5:**
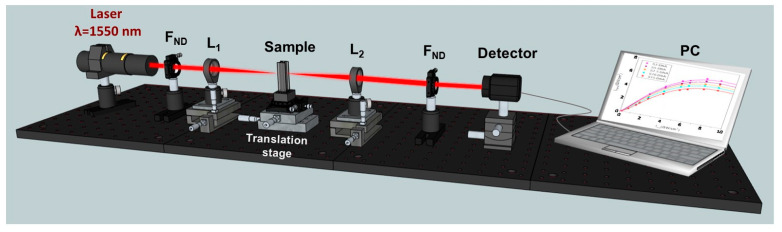
The schematic of the I-scan experimental setup used to investigate the optical limiting potential of DNA biopolymer functionalized with spirulina dye.

A schematic of the I-scan setup used in our experiments is shown in Figure 5.

In our experiments, we used as excitation source an Er-doped fibre laser, FemtoFiber Scientific FFS (TOPTICA Photonics AG, Munich, Germany) at the wavelength λ = 1550 nm, which generates ultrashort pulses with the duration *τ* ~120 fs at a repetition rate of *f*_rep_ = 76 MHz. The maximum average power of the beam incident on the sample was *P*_av_ = 193 mW. At this average power, the laser pulses have a peak power *P*_peak_ = 18.65 kW, considering a sech^2^ temporal profile of the laser pulse. The corresponding pulse energy was 2.54 nJ. The laser power incident on the investigated samples was controlled using different neutral density filters (F_ND_) from Thorlabs (Munich, Germany), specially calibrated by us at λ = 1550 nm. It should be noted that no optical damage was observed in the investigated samples within the entire range of incident beam intensities used in our experiments.

The investigated samples were positioned in the focal plane of the converging lens L_1_, which had a focal length of 2.54 cm. This lens focused the incident beam into a beam with a diameter (full width at half maximum—FWHM) of 13 μm on the sample plane. A converging lens L_2_ with a focal length of 3.5 cm was used to adjust the spot size of the transmitted laser beam to that of the sensitive area of the detector, thus ensuring the measurement of the entire transmitted light.

The cuvette with solution and L_1_, L_2_ lenses was disposed on micrometric translation stages, which allowed precise adjustments of their positioning. The powers of the incident and transmitted beams were corrected, taking into account the appropriate Fresnel reflections on the faces of the L_1_ lens, on the external (air-quartz) and internal (quartz-solution) interfaces of the cuvette, and on the faces of the L_2_ lens.

The incident beam and the beam transmitted through the samples were both measured using the OP-2IR sensor (Coherent, Portland, OR, USA), connected to a FieldMax II-TOP powermeter (Coherent, Portland, OR, USA). Neutral density filters (F_ND_) from the same Thorlabs set of calibrated filters were also placed in front of the detector to ensure that the power of the light incident on the detector remained below its maximum measurable power.

The values of the peak intensity, *I*_peak_, of the beam incident on the sample and of the beam transmitted through it were computed from the values of the corresponding average powers, measured directly with the powermeter. Considering a Gaussian transversal spatial profile and a sech^2^ temporal profile of the ultrashort laser pulses, the analytical expression for the conversion from the average power *P*_av_ to the peak intensity *I*_peak_ was [62]:

(1) $$I_{peak}=2\cdot P_{peak}/(\pi w_{0}^{2})=2\cdot \left(0.88\cdot \frac{P_{av}}{\tau \cdot f_{rep}}\right)/(\pi w_{0}^{2})$$

where *w*_0_ is the waist of the laser beam (radius of the incident laser beam focused with the lens L_1_, at 1/e^2^ from its maximum value at the centre of the beam). The waist *w*_0_ is related to the *FWHM* of the beam by [62]:

(2) $$w_{0}=FWHM/\sqrt{2\ln2}$$

## 3. Results and Discussion

To assess the optical limiting potential of Spirulina natural dye and of DNA–CTMA–spirulina compound by I-scan, we experimentally measured the dependence of the transmitted peak intensities, *I*_peak(trans)_, on the incident peak intensities, *I*_peak(inc)_, for the entire range of *I*_peak(inc)_ of the laser source. The deviation of this dependence from linearity is a measure of the optical limiting potential. A larger deviation towards a constant value of *I*_peak(trans)_ indicates better optical limiting.

**Figure 6:**
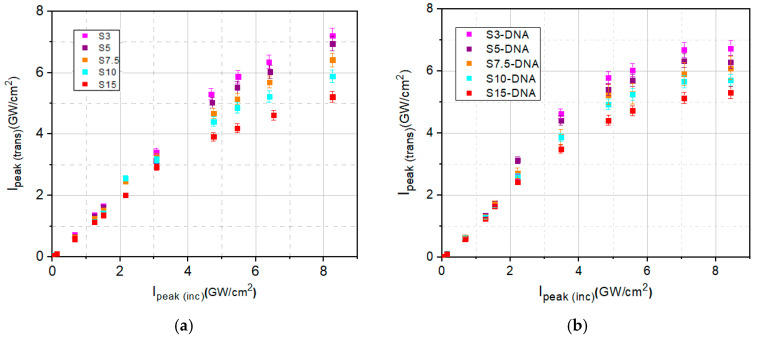
The transmitted peak intensities as function of the incident peak intensities, for all prepared solutions: (**a**) Spirulina solutions in butanol (S3 to S15) and (**b**) DNA-CTMA-spirulina solutions in butanol (S3-DNA to S15-DNA).

The experimental dependencies in the investigation of the optical limiting, *I*_peak(trans)_ on the incident peak intensities, *I*_peak(inc)_, for each investigated sample, without and with DNA–CTMA, are shown in Figure 6 and discussed below.

In Figure 6, it can be seen that the transmitted peak intensities, *I*_peak(trans)_, follow a linear dependence on incident peak intensities, *I*_peak(inc)_, for the low values of the incident ones. From the slope of this linear dependence and taking into consideration the Beer–Lambert law for the linear absorption (Equation (3)):

(3) $$I_{peak\left(trans\right),linear}=I_{peak\left(inc\right)}\cdot e^{-\alpha_{0}\cdot L}$$

where *α*_0_ is the linear absorption coefficient and *L* is the thickness of the sample (equal to the internal thickness of the cuvette), the value of *α*_0_ can be computed as:

(4) $$\alpha_{0}=-\left(1/L\right)\cdot \ln\left(I_{\mathrm{peak}\left(\mathrm{trans}\right),linear}/I_{\mathrm{peak}(\mathrm{inc})}\right)$$

**Figure 7:**
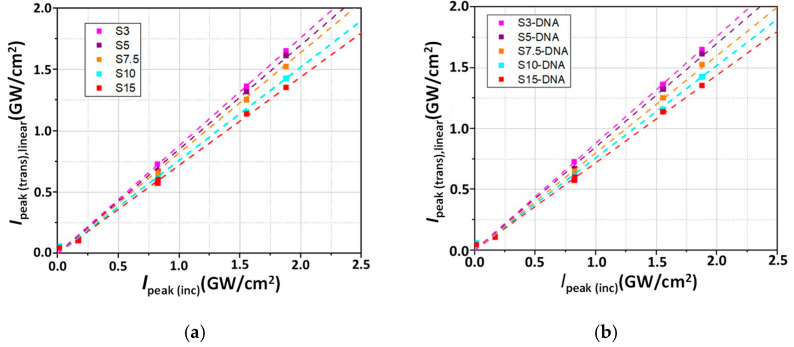
The linear dependences (dashed lines) of the transmitted peak intensities as function of the incident peak intensities, for all prepared solutions, at low incident intensities: (**a**) Spirulina solutions in butanol (S3 to S15) and (**b**) DNA–CTMA–spirulina solutions in butanol (S3-DNA to S15-DNA).

The linear dependencies of the transmitted peak intensities *I*_peak(trans),linear_ on incident peak intensities, *I*_peak(inc)_, at low values of the incident ones, for all investigated spirulina and DNA–CTMA–spirulina solutions are shown in Figure 7.

Using Equation (4) and the data from Figure 7, the linear absorption coefficient, *α*_0_, of each investigated sample has been computed. The obtained values are indicated in Table 2.

The linear dependence of *I*_peak(trans),linear_ on *I*_peak(inc)_ for the low values of the incident ones, is followed by a nonlinear dependence at higher incident intensities, the transmitted peak intensity *I*_peak(trans),NL_ having a nonlinear dependence on *I*_peak(inc)_. This nonlinear dependence indicates a dependence of the absorption coefficient on the incident intensity, *α* = *f*(*I*_peak(inc)_), so that the absorption coefficient no longer has the constant value *α*_0_. Therefore, for high values of incident peak intensities, Equation (3) becomes Equation (5):

(5) $$I_{peak\left(trans\right),NL}=I_{peak\left(inc\right)}\cdot e^{-\alpha \left(I_{peak\left(inc\right)}\right)\cdot L}$$

In our experiments, the dependencies of *I*_peak(trans),NL_ on *I*_peak(inc)_ show a saturation trend. Taking into account a saturation intensity, *I*_sat_, and a nonlinear absorption coefficient, *β*, we considered the following equation for *α*(*I*_peak(inc)_) [11,62], in which both two-photon absorption, specific to high intensity femtosecond pulses [63] and reverse saturable absorption, which can appear due to cumulative effects induced by high-repetition-rate laser pulses [11] are considered:

(6) $$\alpha \left(I_{peak\left(inc\right)}\right)=\frac{\alpha_{0}}{1+\frac{I_{peak\left(inc\right)}}{I_{sat}}}+\beta \cdot I_{peak\left(inc\right)}$$

When *I*_peak(inc)_ decreases, attaining very low values, the value of *α*(*I*_peak(inc)_) tends to the value of the linear absorption coefficient, *α*_0_.

**Figure 8:**
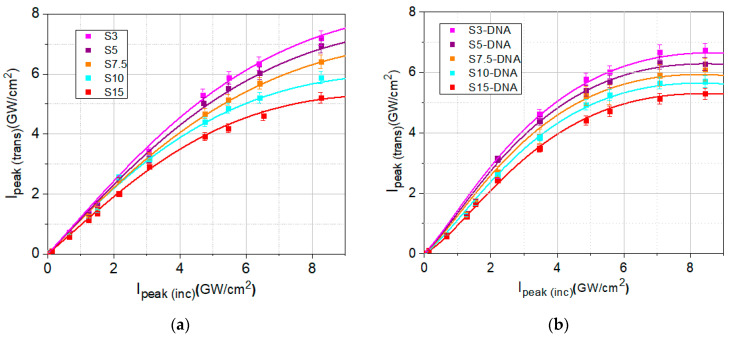
The transmitted peak intensities as function of the incident peak intensities, fitted with Equation (5) in which Equation (6) has been taken into account (solid lines), for all prepared solutions: (**a**) Spirulina solutions in butanol (S3 to S15) and (**b**) DNA–CTMA–spirulina solutions in butanol (S3-DNA to S15-DNA).

The two entire sets of experimental data (dots), shown in Figure 8a,b, have been fitted with Equation (5), taking into account Equation (6) (solid lines). From this fitting the values of *I*_sat_ and *β* have been determined. The values of these parameters are indicated in Table 2.

In Table 2, the values of linear and nonlinear optical parameters (linear transmittance, *T*_L_, linear absorption coefficient, *α*_0_, saturation intensity, *I*_sat_, and nonlinear absorption coefficient, *β*) determined by fitting the experimental data from Figure 7 and Figure 8 are presented.

**Figure 9:**
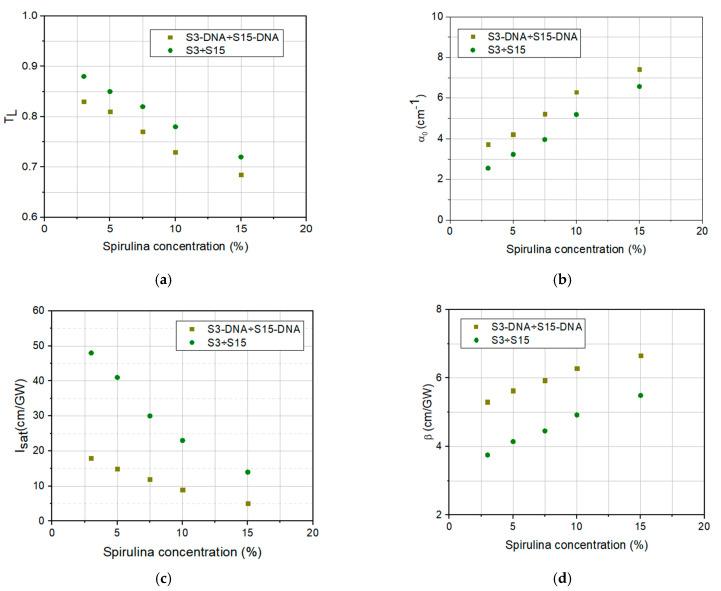
The dependences on spirulina concentration of the linear transmittance, *T*_L_ (**a**), linear absorption coefficient, *α*_0_ (**b**), saturation intensity, *I*_sat_ (**c**), and the nonlinear absorption coefficient, *β* (**d**), of the investigated spirulina solutions and DNA–CTMA–spirulina solutions.

The values of *T*_L_, *α*_0_, *I*_sat_, *β*, from Table 2, obtained from the fitting of the recorded experimental data from Figure 7a,b and Figure 8a,b, are graphically represented in Figure 9a–d. These data highlight the influence of DNA–CTMA on the linear and nonlinear optical absorption properties of DNA–CTMA–spirulina solutions, compared to those of spirulina solutions, at λ = 1550 nm wavelength, and also the influence of the spirulina concentration on the same properties.

The analysis of the data from Table 2 and Figure 9a–d reveals that not only the linear absorption coefficient *α*_0_ is dependent on the spirulina concentration in the two sets of samples investigated, but also the parameters that quantify their nonlinear response, *I*_sat_, and *β*. It is possible to see from the table that both the linear and nonlinear absorption coefficients, *α*_0_ and *β*, respectively, increase when the concentration of spirulina increases. An opposite trend appears for the saturation intensity *I*_sat_, which decreases when the spirulina concentration increases. The DNA–CTMA influences the optical limiting positively and significantly, *β* being larger and *I*_sat_ lower in the samples containing it, than in those with spirulina only. The linear absorption coefficient *α*_0_ is also increased in samples with DNA–CTMA compared to those with spirulina only, leading to lower linear transmittance. The differences between the values of *T*_L_, *α*_0_, *I*_sat_, and *β* parameters in DNA–CTMA–spirulina samples and in spirulina alone samples slightly diminish with the increase in dye concentration, in the considered concentration range.

A comparison of the values of *T*_L_ and *α*_0_ parameters for both sets of the investigated samples with the values of the same parameters of water at λ = 1550 nm wavelength, *T*_L,water_ = 0.67046 (at the same thickness, 0.5 mm) and *α*_0,water_ = 7.9959 cm^−1^ [64], reveals that all investigated samples have low linear absorption, being even more transparent than water, a positive fact in their use as optical limiting materials in NIR [65]. Concerning the values of the nonlinear absorption coefficient, *β*, obtained in this study, a comparison with the values of the same parameter for several other natural dye extracts is shown in Table 3.

Analysing the data presented in Table 3, it is possible to see that the values of the nonlinear absorption coefficient, *β*, for the investigated solutions, with and without DNA–CTMA, are either comparable or better than the corresponding values for other natural dye extracts, reported in other studies in the literature [15,66,67]. Also, as reported in the present work and in [15], the values of the nonlinear absorption coefficient, *β*, are larger in compounds containing the DNA–CTMA biopolymer. The DNA biopolymer can favourably influence the optical response of the dye in several ways: prevention of the aggregation of dye molecules, which is responsible for the intensity decrease in many optical properties [28], protection against photodegradation and thermal degradation of dye molecules [15], change in the local environment for the dye molecules [28].

Thus, the obtained results reveal the beneficial influence of the DNA on the optical limiting in the novel DNA–CTMA–spirulina compound.

## 4. Conclusions

The passive optical limiting in the spirulina natural dye and in the novel DNA–CTMA–spirulina compound has been experimentally demonstrated, for the first time to the best of our knowledge.

The results of an experimental comparative study on absorptive nonlinear optical properties of DNA–CTMA functionalized with spirulina of different concentrations, as solutions in butanol, and on the same nonlinear optical properties of similar solutions with spirulina alone have been presented. The experimental investigation of the absorptive nonlinear optical response was performed by the I-scan method using near-infrared ultrashort laser pulses (~120 fs) at the wavelength of 1550 nm.

Important linear and nonlinear optical parameters (linear absorption coefficient, saturation intensity, and nonlinear absorption coefficient) have been determined in the investigated materials, assessing the optical limiting potential of DNA–CTMA–spirulina and of spirulina dye alone and the effect of DNA on the nonlinear optical response. The performed study demonstrated the beneficial effect of DNA biopolymer on the nonlinear optical response of the investigated solutions, improving their nonlinear optical properties and optical limiting.

The presented results revealed the potential of DNA biopolymer–natural dye compound for photonic applications, in particular optical limiting, serving as a “green” alternative to synthetic polymers and dyes.

## Figures and Tables

**Table 1:** The investigated solutions (spirulina in butanol and DNA–CTMA–spirulina in butanol) and their spirulina concentration.

Denomination of Samples	Spirulina Concentration in Solution g/L
S3/S3-DNA	3
S5/S5-DNA	5
S7.5/S7.5-DNA	7.5
S10/S10-DNA	10
S15/S15-DNA	15

**Table 2:** The values of the linear transmittance, *T*_L_, of the linear absorption coefficient, *α*_0_, of the saturation intensity, *I*_sat_, and of the nonlinear absorption coefficient, *β*, determined from the fitting of the experimental data (Figure 7 and Figure 8), for samples S3 to S15 and S3-DNA to S15-DNA.

Sample	*T*_L_	*α*_0_ (cm^−1^)	*I*_sat_ (GW/cm^2^)	*β* (cm/GW)
S3/S3-DNA	0.88/0.83	2.55/3.73	48/18	3.75/5.30
S5/S5-DNA	0.85/0.81	3.25/4.21	41/15	4.14/5.63
S7.5/S7.5-DNA	0.82/0.77	3.96/5.22	30/12	4.45/5.93
S10/S10-DNA	0.77/0.73	5.22/6.29	23/9	4.92/6.28
S15/S15-DNA	0.72/0.69	6.57/7.42	14/5	5.49/6.63

**Table 3:** Comparison of the values of the nonlinear absorption coefficient, *β,* obtained in the present work with the values of several other natural dye extracts reported in the literature.

Nonlinear Compound/Dye	*β* (cm/GW)	References
Turmeric–DNA/Turmeric	4/3.4	[15]
Spinach (Violxanthine dye)	1.87	[66]
Hibiscus Sabdariffa	2.1 × 10^−4^	[67]
Spirulina–DNA/Spirulina	6.63/5.49	present work

## Data Availability

Data are contained within the article.
